# Load-Independent Hardness and Indentation Size Effect in Iron Aluminides

**DOI:** 10.3390/ma17092107

**Published:** 2024-04-29

**Authors:** Sebastian Balos, Milan Pecanac, Mirjana Trivkovic, Savo Bojic, Pavel Hanus

**Affiliations:** 1 Department of Production Engineering, Faculty of Technical Sciences, University of Novi Sad, Trg Dositeja Obradovica 6, 21000 Novi Sad, Serbia; sebab@uns.ac.rs (S.B.); mirjana.trivkovic@uns.ac.rs (M.T.); savobojic@uns.ac.rs (S.B.); 2 Department of Material Science, Faculty of Mechanical Engineering, Technical University of Liberec, Studentská 1402/2, 461 17 Liberec, Czech Republic; pavel.hanus@tul.cz

**Keywords:** intermetallic compounds, microhardness, load-independent hardness

## Abstract

In this paper, an iron–aluminide intermetallic compound with cerium addition was subjected to Vickers microhardness testing. A full range of Vickers microhardness loadings was applied: 10, 25, 50, 100, 200, 300, 500, and 1000 g. Tests were conducted in two areas: 0.5 mm under the surface of the rolled specimen and in the center. The aim was to find the optimal loading range that gives the true material microhardness, also deemed load-independent hardness, H_LIH_. The results suggest that in the surface area, the reverse indentation size effect (RISE) occurred, similar to ceramics and brittle materials, while in the center, indentation size effect (ISE) behavior was obtained, more similar to metals. This clearly indicated an optimal microhardness of over 500 g in the surface region and over 100 g in the central region of the specimen. Load dependencies were quantitatively described by Meyer’s law, proportional specimen resistance (PSR), and the modified PSR model. The modified PSR model proved to be the most adequate.

## 1. Introduction

The primary engineering materials utilized today are alloys, which are compounds formed by combining two or more metals with non-metallic elements. Alloys exist in two principal forms: solid solutions and intermetallic compounds (IC) [1]. Solid solutions maintain the crystal structure of the parent metal, with alloying elements manifesting as either substitutional (replacing parent metal atoms) or interstitial (occupying lattice interstices), retaining properties of the parent metal, such as electrical and heat conductivity, ductility, and luster, but, very importantly, with increased strength compared to pure metals. Additionally, alloying elements confer synergistic attributes, such as increased corrosion resistance or enhanced mechanical properties. Conversely, ICs exhibit atomic-level ordering over extended distances, endowing them with distinctive characteristics such as elevated hardness and reduced ductility, alongside favorable high-temperature mechanical properties and chemical stability [2].

These distinctive properties endow ICs with a range of unique applications, including hydrogen storage [2,3], catalysis [4,5], shape memory [6,7], superconductors [8,9], and structural applications [10,11]. Structural applications are especially advantageous when high-temperature-resistant components are required due to the high melting and disordering temperatures, high stiffness, and low diffusivity of ICs [10,11]. Moreover, IC may have also been applicable for ballistic protection due to relatively low density (aluminide ICs), high strain hardening rate, enhanced oxidation and temperature resistance, and some formulations containing minimal or no critical raw materials [12,13]. The determination of the mechanical properties of structural ICs is well covered with standards and procedures. However, it is not fully covered in regard to the most widely used microhardness measurement method, Vickers testing, particularly in terms of the test load. Several studies used different Vickers microhardness test loads (Table 1). 

Kant et al. [14] applied two Vickers loads, with the higher 10 kg load for bulk material testing and 25 g for determining the microhardness of the matrix and different phases of the FeAl IC alloyed with C and Ti. This material was fabricated via an arc melting process in an argon atmosphere. In contrast, while Nayak et al. [15] applied a 300 g load on FeAl IC obtained from nanopowders, consolidated in a hydraulic press at 375 MPa for 15 min to form 12 mm diameter discs, Basariya et al. [16] applied a load of only 5 g for microhardness testing of Fe_2_Al_5_, which was prepared using high-purity Al and Fe elements together via arc melting in an argon atmosphere, followed by homogenization at 1000 °C for 2 h, crushing, and testing. A similar powder fabrication method was employed in another study by Basariya et al. [17], but with the application of a 15 g Vickers microhardness measurement load. Massalski et al. [18], on the other hand, utilized a 1 kg load to measure the full range of different ferroaluminide intermetallic compounds (FeAl, Fe_3_Al, FeAl_2_, Fe_2_Al_5_, and FeAl_3_). 

**Figure 1:**
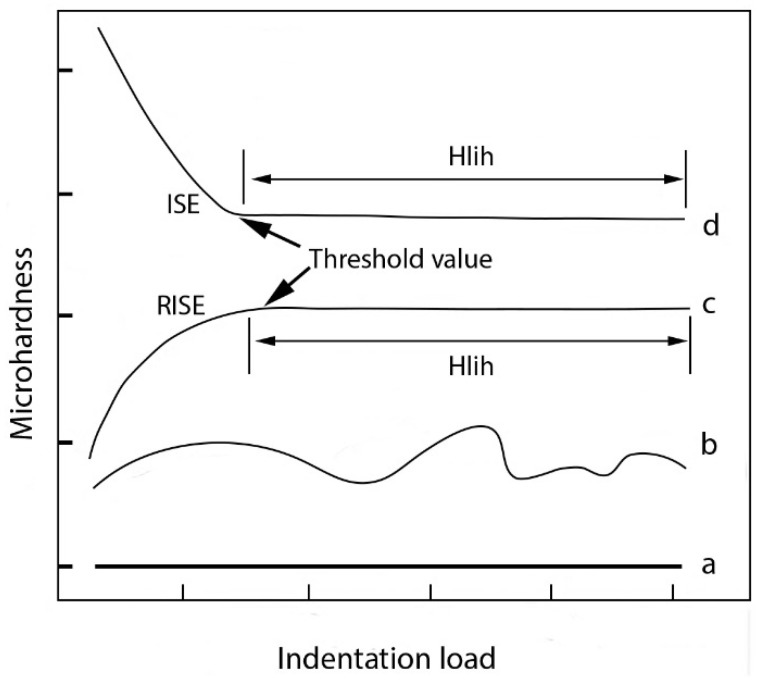
Types of microhardness vs. applied indentation test load behaviors in materials.

Various researchers utilized different microhardness loads—from 5 g to 300 g, without the determination of load-independent hardness (H_LIH_) [19]. This issue can make comparisons difficult and unreliable and can be overcome with consideration of the phenomenon of the indentation size effect (ISE) [20]. The ISE refers to the indentation-depth-dependent hardness, implying that different values of microhardness are obtained by applying different Vickers test loads [21,22]. It must be noted that although both hardness and microhardness measure a material’s resistance to deformation, hardness is measured at a macroscopic scale using larger loads, giving an insight into a material’s bulk mechanical properties, and microhardness is measured at a microscopic scale using smaller loads, providing insights into the material’s microstructure and local mechanical properties. H_LIH_, referring to hardness as a general methodological term, is typically acquired at an indentation load above the threshold. In brittle materials such as certain polymers, relatively high loads can induce cracking, imposing limitations on indentation loads. Four distinct behaviors have been reported thus far, as illustrated in Figure 1: approximately constant microhardness vs. load (Figure 1 line a); irregular values with multiple local maxima and minima (Figure 1 line b); reversed indentation size effect (RISE) in Figure 1, line c; and indentation size effect (ISE) in Figure 1, line d. The constant microhardness vs. load values are observed in the case of ideal instrument and material response conditions [23]. The behavior showcasing irregular values obtained at different indentation loads, shown in Figure 1 line b, is exhibited by some organic crystals and polymers [24]. The RISE behavior, where an increase in test load influences the increased microhardness values up to a threshold load value, resulting in reaching H_LIH_, was found in some metals, obtained via selective laser melting (SLM) and tested via Vickers and Knoop microhardness methods [25,26]. Conversely, brittle polymers and ceramics exhibit ISE behavior (Figure 1, line d), where the rise in test load influences the obtaining of lower microhardness values, again up to a threshold load value, which results in reaching H_LIH_. The ISE effect stands in direct contrast to the RISE [27]. In these materials, cracks may compromise the accuracy of diagonal measurement, thereby affecting the acquired microhardness values [21]. Consequently, both the RISE and the ISE plateau after reaching a critical test load, representing the load-independent hardness. These phenomena may arise due to plastic deformation and dislocation movement, which elevate flow stress and microhardness values [28,29].

The overarching objective of this study is to investigate the indentation behavior of the ferroaluminide intermetallic compound during microhardness testing across a range of loads, with the aim of identifying the optimal loading parameters. Specifically, it is essential to determine the threshold or minimum Vickers microhardness load necessary to ascertain the material’s true microhardness, also referred to as load-independent hardness. Consequently, establishing this loading range would serve as a valuable recommendation for future studies involving similar materials conducted by other researchers.

## 2. Experimental Section

In this study, the tested material was an iron aluminide intermetallic compound (IC) with its chemical composition determined by the titration method (wet chemical analysis) (Table 2). It can be seen that the Fe_3_Al IC is alloyed with Cr and Ce.

**Figure 2:**
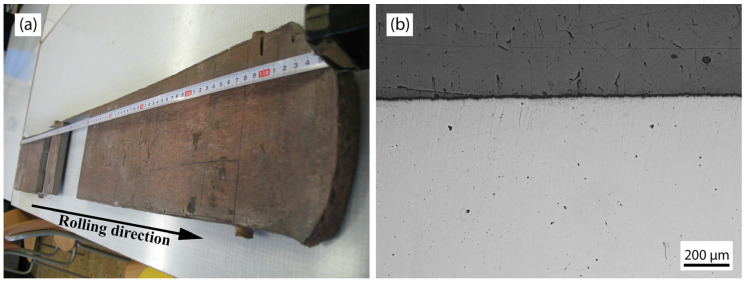
Test piece: (**a**) rolled material; (**b**) polished area under the surface.

The samples for the tests were fabricated through the vacuum melting of the alloy within a shielding argon atmosphere. The molten alloy was then cast into a shell mold, followed by rolling at 1200 °C to achieve half thickness. This procedure has been comprehensively detailed in previous studies conducted by Kratochvil et al. [13,30,31]. The resulting flat work attained a final thickness of 15.2 mm. The visual appearance of the flat work is depicted in Figure 2a, showcasing the entire flat work on the left and a magnified view of the surface area devoid of defects such as cracks, pits, grooves, and irregular surface roughness features (Figure 2b).

Following material fabrication, standard metallographic preparation was performed on Struers laboratory equipment, consisting of a range of steps: cutting with emulsion cooling, mounting in polyethylene cups, grinding using a set of SiC abrasive papers (ranging from grit P100 to P2500), and polishing with 6, 3, 1, and ¼ µm diamond suspensions (Buehler, Lake Bluff, IL, USA). The last step of microstructure preparation involved the utilization of OP-S suspension, which comprises fumed silica with an agglomerated grain size of ¼ µm to minimize the surface deformation of specimens and reduce scratches. Etching was carried out using Rollason solution (100 mL H_2_O + 50 mL 38% HCl + 5 g FeCl_3_) at room temperature (20 °C) and with a duration of 15 s, while microstructural examination was conducted using a Epiphot 200 (Nikon, Konan, Minato, Japan) light microscope with Nomarski differential interference contrast. Cerium particles were observed using a Mira 3 XMH (Tescan, Brno, Czech Republic) scanning electron microscope (SEM), operating at 20 kV, equipped with an Ultim Max 65 (Oxford Instruments, Abingdon, UK) energy-dispersive detector (EDS).

Microhardness measurements were conducted on ground surfaces in separate specimens, using a set of SiC abrasive papers (beginning with grit P100, to P2500). Subsequent polishing was conducted using 6, 3, 1, and ¼ µm diamond suspensions. Before microhardness measurement, specimen surfaces were checked via a Orthoplan (Leica–Leitz, Wetzlar, Germany) light microscope. Microhardness was measured with a Wilson Tukon 1102 (Buehler, Lake Bluff, IL, USA) device, using various loads: 10, 25, 50, 100, 200, 300, 500, and 1000 g. Measurements were conducted at two locations: at the center of the specimen and 0.5 mm beneath the surface. Each measurement was repeated three times for every applied load. Indentations were observed using a JSM-6460LV (JEOL Ltd., Tokyo, Japan) scanning electron microscope (SEM) operating at 20 kV. Prior to observation, specimens were coated with gold using a Bal-tec SCD-005 (Leica–Leitz, Wetzlar, Germany) sputter coating device. 

The load-dependence of the measured Vickers microhardnesses was quantitatively described through the application of the conventional Meyer’s law, proportional specimen resistance (PSR), and the modified PSR model [23].

The first attempt to describe the dependence of the indentation load and average measured Vickers indentation diagonal is known as Meyer’s law. This dependence has the following exponential form:

P = Ad^n^, (1)

where P is the indentation load, d is the indentation size obtained as the average of two measured diagonals, and parameters A and n are values that are obtained from the curve fitting of the experimental data.

An attempt to create a more accurate model compared to Meyer’s law of load-indentation size is represented by the PSR model based on a polynomial equation of the second order:

P = a_1_d + a_2_d^2^, (2)

where a_1_ and a_2_ are experimental constants, P is the indentation load, and d is the indentation size.

The modified PSR model was proposed by Gong and Li [23]. They found that specimen preparation in the form of grinding and polishing induces surface stress that can influence the size of the indentation and therefore the obtained microhardness results. The modified PSR model adds an experimental constant Po:

P = P_o_ + a_1_d + a_2_d^2^. (3)

## 3. Results and Discussion

**Figure 3:**
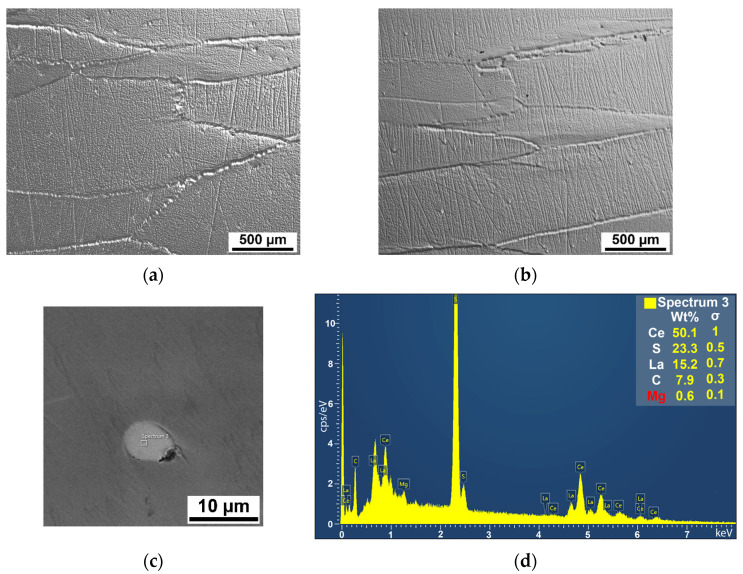
Microstructure of the flat test piece: (**a**) in the central part of the thickness; (**b**) close to surface; (**c**) Ce-based particle; (**d**) EDS results of complex Ce-based particles.

The microstructures in the longitudinal direction (rolling direction along the tape measure, as depicted in Figure 2) of the tested sample are shown in Figure 3. In the central part of the thickness, the visible deformation texture of large, elongated grains is observed, which arises as a consequence of the rolling process. The grain boundaries exhibit waviness, indicative of dynamical and post-dynamical recovery processes during rolling (Figure 3a). In the area close to the surface of the sample (Figure 3b), the grains appear elongated due to the heightened local pressure experienced in the rolling mill, particularly localized compressive stresses. The recovery process of grains is less pronounced in this region, primarily due to the efficient heat dissipation from the sample surface by the cold cylinder(s) in the rolling mill. This cooling process reduces the kinetics of recovery processes within the surface zone.

The results of EDS analysis are depicted in Figure 3c,d. Complex cerium (Ce) particles, with a size of approximately 5 µm, are uniformly dispersed throughout the entire area, residing within grains as well as along their boundaries. The microparticles, measuring roughly 5 µm in size, also contain sulfur (S), lanthanum (La), carbon (C), and a minute quantity of magnesium (Mg).

**Figure 4:**
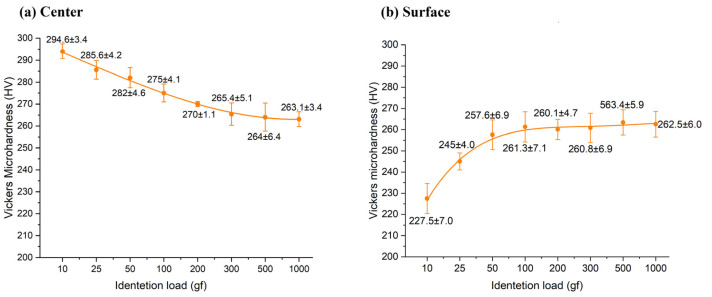
Vickers microhardness in relation to indentation load: (**a**) center (ISE); (**b**) surface (RISE).

The results of measured Vickers microhardness versus indentation load are presented in charts within Figure 4. It is apparent that microhardness values exhibit different trends: a distinct ISE in the specimen center (Figure 4a) and RISE in surface measurements (Figure 4b). At lower indentation loads, there are significant differences in microhardness values: 227.5 and 294.6 HV at 10 g indentation load for surface and center areas, respectively. Afterwards, both analyzed areas (surface and specimen center) exhibit a trend whereby microhardness values of approximately 260 HV are reached at higher indentation loads, which is a typical response in accordance with Figure 1.

The RISE observed in the surface layer mirrors that commonly found in ceramics, characteristic of typical brittle materials. This behavior can be attributed to the compressed surface area resulting from the rolling fabrication process. Additionally, there is a proposition suggesting that hydrogen atoms occupy interstitial sites within the Fe_3_Al intermetallic compound (IC), leading to environmental embrittlement in the surface region. This phenomenon renders the IC’s behavior akin to that of ceramics, as elucidated by Stoloff and Liu [32]. 

The values approaching 260 HV, forming a plateau, signify the region of load-independent hardness, which is attained at different loads in two distinct measurement locations. These loads serve as threshold values beyond which H_LIH_ is achieved. In the center of the specimen, the load-independent hardness value is reached after approximately 500 g load, whereas in the surface region, it is reached after 100 g. Consequently, it is advisable to measure the load-independent hardness of the intermetallic compound at loads exceeding 500 g, indicating that the microhardness falls within the range of 260 to 265 HV. 

**Figure 5:**
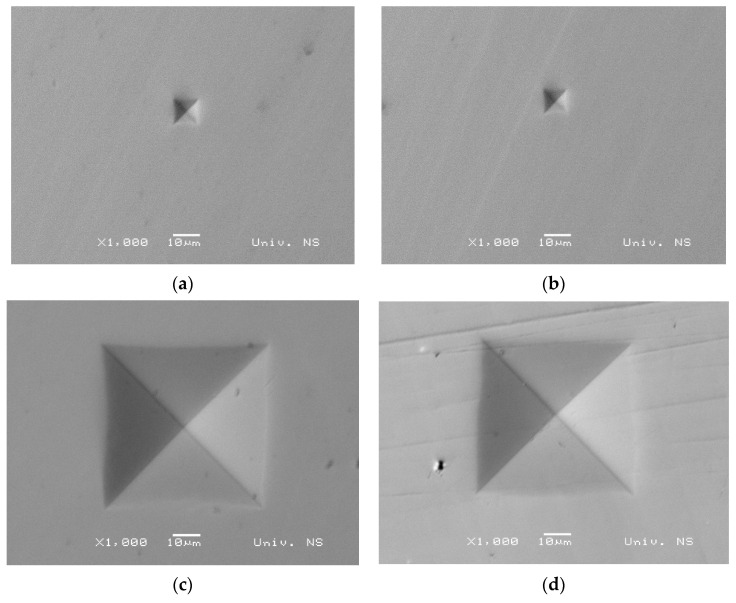
Indentations: (**a**) 25 g under the specimen surface; (**b**) 25 g in the specimen center; (**c**) 1 kg under the specimen surface; (**d**) 1 kg in the specimen center.

SEM images of indentations are shown in Figure 5. It can be seen that a near-perfect square shape is obtained at higher indentation loads. At high indentation loads, a slight distortion is present, which is the result of elastic recovery during the unloading and the removal of the indenter, as found by Trzepiecinski and Lemu [33]. However, at lower indentation loads, a slight sink-in phenomenon is observed, resulting in a minor concavity of the indentation, typical for elastic materials [34]. It is noteworthy that no cracks were observed during microhardness testing, which do occur in brittle materials such as ceramics, as reported by Muchtar et al. [35], and certain types of polymers such as Poly(methyl methacrylate), as found by Balos et al. [20].

**Figure 6:**
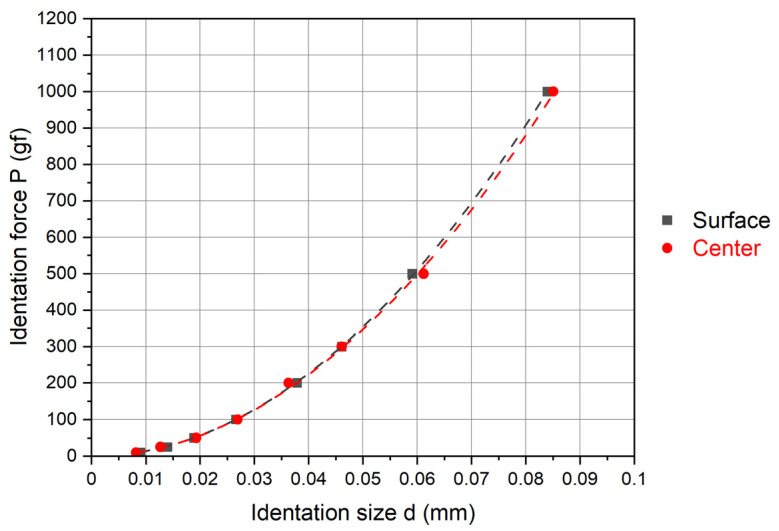
Correlation between indentation load (P) and average indentation diagonal (d) via Meyer’s law for specimen surface and specimen center measurements.

**Figure 7:**
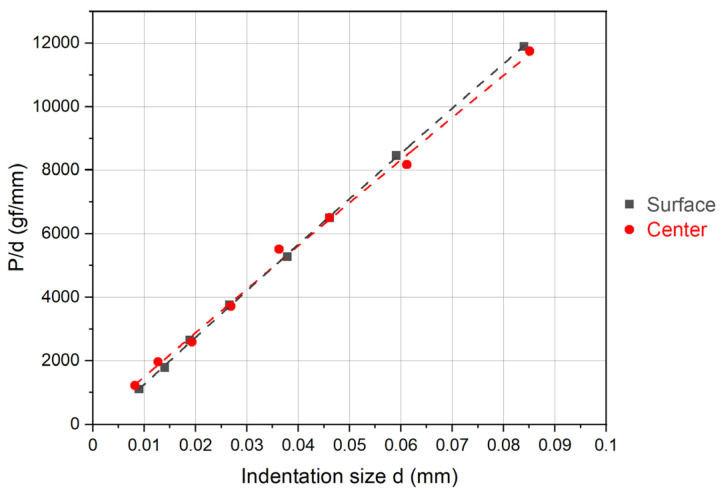
Correlation between indentation load (P) and average indentation diagonal (d) via the PSR model for surface and center measurements.

**Figure 8:**
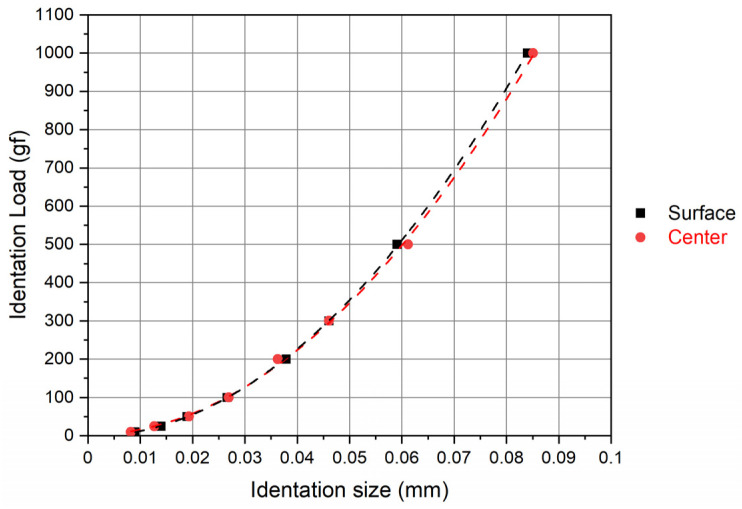
Correlation between indentation load (P) and average indentation diagonal (d) via the modified PSR model for surface and center measurements.

The dependencies of indentation size on load are quantitatively described through the application of Meyer’s law (Figure 6), PSR (Figure 7), and the modified PSR model (Figure 8). Additionally, correlation factors (R^2^) are presented alongside the dependency trends in these figures. A correlation factor closer to 1 indicates a better fit of the mathematical model to the obtained results. Table 3, Table 4 and Table 5 present the results of regression analyses for Meyer’s law, PSR, and the modified PSR model, derived from the logarithmic (Meyer) and polynomial (PSR and modified PSR) trendlines displayed in Figure 6, Figure 7 and Figure 8. Notably, the power law exponent is higher in specimens where the microhardness was measured on the specimen surface area, indicating a more pronounced indentation size effect (ISE) in this region compared to the central area. This finding aligns with the Vickers microhardness-to-indentation-load curves illustrated in Figure 4.

Figure 7 and Table 4 illustrate the correlation between indentation load (P) and average indentation diagonal (d) and the results of the regression analysis using the PSR model. It is notable that the correlation factor (R^2^) associated with surface microhardness is higher compared to that obtained with Meyer’s law. Conversely, the correlation factor (R^2^) pertaining to centerline measurements is higher in the Meyer’s-law-analyzed indentations, suggesting that Meyer’s law is more appropriate in describing the dependencies of indentation size on load.

The correlation between indentation load (P) and average indentation diagonal (d), as well as correlation factors (R^2^) for the surface and center of the intermetallic compound specimen in accordance with the modified PSR model, are presented in Figure 8 and Table 5. The modified PSR model was suggested by Gong and Li [23] and differs from the PSR model by taking into account the influence of surface features of the specimen, which refers to specimen preparations such as grinding and polishing, which are necessary for Vickers microhardness measurements. It is evident that the surface layer measurements exhibit the highest correlation factor (R^2^) of all the models used, indicating that the modified PSR model is the most adequate for describing indentation-load-to-indentation-size dependencies. Conversely, measurements conducted in the central part of the specimen exhibited a correlation factor of 0.9992, which is higher and thus more adequate than that achieved by the PSR model and equal to the values obtained by applying Meyer’s law. These results are similar to those obtained by Gong et al. [23], as well as Balos et al. [20,25,26], where an added experimental constant P_o_ was found to enhance the accuracy of the model, irrespective of the tested material, i.e., polymers or alloys, obtained as bulk material or additively manufactured, and tested by the Vickers or the Knoop microhardness testing method.

## 4. Conclusions

Based on the findings of this study, and acknowledging its limitations, the following key conclusions can be drawn:

- Vickers microhardness demonstrates a significant dependency on both the applied indentation load and the specific measurement location.
- In the surface region, a clear reversed indentation size effect (RISE) behavior was observed, characterized by a plateau reached at a 100 g indentation load. In contrast, in the center of the specimen, an indentation size effect (ISE) behavior was identified, with relatively constant microhardness values achieved at a higher load of 500 g, meaning that the recommended indentation load for obtaining load-independent hardness is 500 g or more.
- Lower indentation loads result in a slight concavity of the indentation, indicative of elastic material behavior. However, at higher loads, a slight distortion is observed across all measurements, without any instances of cracking.
- Meyer’s law, proportional specimen resistance (PSR), and modified PSR models have all proven highly adequate in describing the relationship between indentation load and size. Notably, the modified PSR model demonstrated the highest correlation factors among the models tested.

## Figures and Tables

**Table 1:** Microhardness loads applied in various studies.

IC Composition	Vickers Load	Reference
FeAl + (0.1; 1)% C, (0; 1; 5)%Ti	25 g	[14]
FeAl	300 g	[15]
Fe_2_Al_5_	5 g	[16]
FeAl_3_, FeAl_2_	15 g	[17]
FeAl	100 g	[18]

**Table 2:** Chemical composition of the iron aluminide intermetallic material [mass. %].

Al	Cr	Ce	Fe
16.53	2.7	0.02	Balance

**Table 3:** Regression analysis of the experimental data in accordance with Meyer’s law.

	A	N	R^2^
Surface	170.30	2.0617	0.9997
Center	124.29	1.9617	0.9992

**Table 4:** Regression analysis of the experimental data in accordance with the PSR model.

	a1	a2	R^2^
Surface	140,769	−61,145	0.9998
Center	139,896	−47,978	0.9963

**Table 5:** Regression analysis of the experimental data in accordance with the modified PSR model.

	Po	a1	a2	R
Surface	−3.1383	96.551	141.182	1
Center	1.1444	129.35	135.667	0.9992

## Data Availability

Data are contained within the article.
